# New benchmarks in the modelling of X-ray atomic form factors

**DOI:** 10.1107/S2053273323003996

**Published:** 2023-06-02

**Authors:** Gunnar Thorkildsen

**Affiliations:** a Department of Mathematics and Physics, University of Stavanger, N-4036 Stavanger, Norway; Institute of Crystallography - CNR, Bari, Italy

**Keywords:** atomic form factors, analytical representations, inverse Mott–Bethe formula

## Abstract

Analytical representations of X-ray atomic form factor data have been determined. The original data, *f*_0_(*s*;*Z*), are reproduced to a high degree of accuracy. The mean absolute errors calculated for all *s* = sin θ/λ and *Z* values in question are primarily determined by the precision of the published data. The inverse Mott–Bethe formula is the underlying basis with the electron scattering factor expressed by an expansion in Gaussian basis functions. The number of Gaussians depends upon the element and the data and is in the range 6–20. The refinement procedure, conducted to obtain the parameters of the models, is carried out for seven different form factor tables published in the span Cromer & Mann [(1968), *Acta Cryst.* A**24**, 321–324] to Olukayode *et al.* [(2023), *Acta Cryst.* A**79**, 59–79]. The *s* ranges are finite, the most common span being [0.0, 6.0] Å^−1^. Only one function for each element is needed to model the full range. This presentation to a large extent makes use of a detailed graphical account of the results.

## Abstract 2

Improved analytical representations of X-ray atomic form factors are put forward based on the inverse Mott–Bethe formula. Applying these representations, the mean absolute errors calculated for the complete set of form factors given in Table 6.1.1.1 in *International Tables for Crystallography*, Vol. C, 3rd ed., are reduced by a factor of ∼50 from previous published analyses. Various form factor compilations are examined to record the applicability of the approach outlined.

## 1. Introduction

Calculations of X-ray atomic form factors, 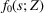 , where 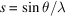 ( 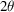 is the angle between the incoming and scattered wavevectors, λ is the wavelength in question) and *Z* is the atomic number, have always followed in the wake of the ongoing development within quantum mechanics and numerical/computational methods. Thus extensive tables of 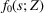 have been frequently published in the crystallographic literature. Various analytical expressions, *i.e.* functions in the independent variable *s*, have been examined to model the tabulated data and ease their use in various calculations. Parameters entering these functions are determined by least-squares procedures which sometimes involve specific weight schemes. Early papers by Vand *et al.* (1957 ▸), Forsyth & Wells (1959 ▸) and Moore (1963 ▸) give parameters in Gaussian models associated with form factor calculations by James & Brindley (1931*a* ▸,*b* ▸), Viervoll & Ögrim (1949 ▸), McWeeny (1951 ▸), Hoerni & Ibers (1954 ▸), Berghuis *et al.* (1955 ▸), Thomas & Umeda (1957 ▸), Freeman & Smith (1958 ▸) and Freeman (1959 ▸), *cf*. Ibers (1962 ▸). Papers of greater impact for the present work are summarized in Table 1 ▸. To fill out the picture, one should also consult the works by Onken & Fischer (1968 ▸), Lie (1977 ▸), Weickenmeier & Kohl (1991 ▸), Peng *et al.* (1996 ▸), Szalóki (1996 ▸), Su & Coppens (1998 ▸), Macchi & Coppens (2001 ▸), Feranchuk *et al.* (2002 ▸) and Muhammad & Lee (2013 ▸). For extensive sets of X-ray atomic form factor data, the reader is advised to look up the works of Hubbell *et al.* (1975 ▸), Hubbell & Øverbø (1979 ▸), databases EPDL97 (Cullen *et al.*, 1997 ▸), RTAB (Kissel, 2000 ▸), EPICS2017 (Cullen, 2018 ▸), and the software environments *XOP* (Sánchez del Río & Dejus, 1997 ▸, 2011 ▸) and *XRAYLIB* (Brunetti *et al.*, 2004 ▸; Schoonjans *et al.*, 2011 ▸).

In this work, cases where *s* spans a finite interval, *e.g.* 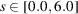 Å^−1^, are addressed. Thus characteristic asymptotic properties in the limit 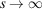 are not taken into consideration. This also warrants the inclusion of refinable constants such as *c* and α in equations (3) and (5) below.

**Figure 1:**
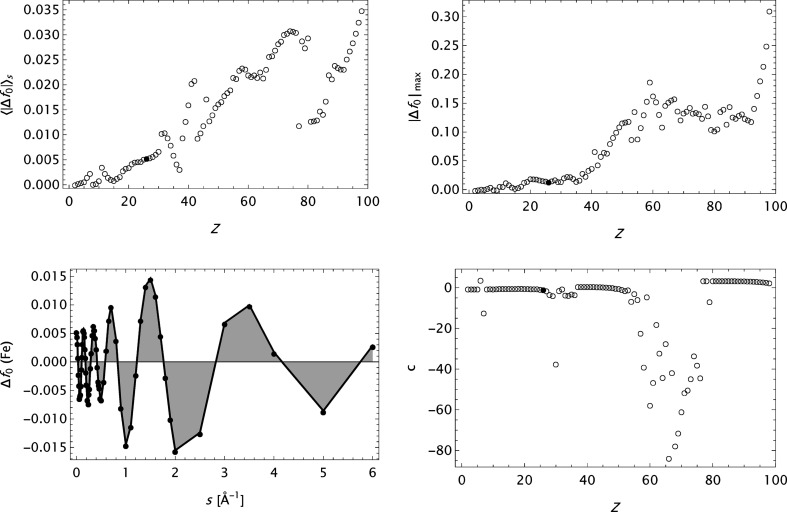
Summary of some results based on the analytical model 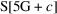 (Waasmaier & Kirfel, 1995 ▸) applied to the X-ray form factor data in *International Tables for Crystallography* Vol. C, 1st ed. Iron (Fe, *Z* = 26) is indicated by a filled circle, the other elements by empty circles. Upper left: mean absolute error 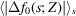 as a function of the atomic number *Z*. Upper right: maximum absolute error 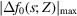 as a function of the atomic number *Z*. Lower left: the error (or deviation) 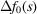 calculated for iron. Lower right: variation of the parameter *c* with the atomic number *Z*.

The main reference for the present analysis is the form factor data presented in Table 6.1.1.1 in *International Tables for Crystallography*, Vol. C (Maslen *et al.*, 1992 ▸), and the analytical modelling by a five-Gaussian expansion (Waasmaier & Kirfel, 1995 ▸). Some key features herein are summarized in Fig. 1 ▸. The mean and maximum absolute errors 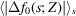 and 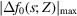 are presented as functions of *Z* { 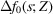 = 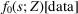 − 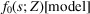 }. Furthermore, it is also shown that 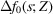 exhibits an oscillating behaviour as a function of *s*, here depicted for *Z* = 26 (Fe). This signature is relatively insensitive to the value of *Z* and it is assumed to be primarily associated with inherent features of the quantum mechanical calculations. The most frequently used analytical model, the *n*-Gaussians expression, is apparently not capable of modelling such a behaviour. Finally, the large irregular variation in the parameter *c* as a function of *Z* is noted.

Table 1 in the paper by Waasmaier & Kirfel (1995 ▸), *Parameters of analytical scattering-factor functions* (*a*) *For neutral atoms*, has no explicit ordering of parameters. It is advisable to arrange the *b*_1_–*b*_5_ parameters in increasing order with a subsequent rearrangement of the *a* parameters. Such an ordering may help in revealing any challenges, but also systematic trends across the Periodic Table. This gives support when initial values for the parameters in the least-squares treatment are to be selected. For the elements 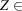 {18, 38–42, 46, 78, 80} two out of the five *b* parameters have (almost) equal values. Subsequently, building the normal matrix of the least-squares calculation in these cases may result in a non-positive-definite matrix and thus prevent uncertainty assessments. For most elements the *c* parameter has a value close to zero, but especially in the range 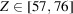 one observes large negative values. A maximum magnitude of 83.3 is found for *Z* = 66 (Dy). A separate review of this case, *cf*. Table 2 ▸, demonstrates that 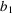 has a very small value, making 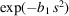 almost unity across the actual span in *s*. The corresponding coefficient, 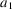 , is approximately equal to 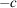 . Their sum amounts to the true constant in the model. A signature of both quantities is the anomalously large magnitudes. The uncertainties, { 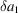 , 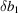 , 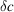 }, considerably exceed 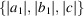 , so these parameters are in practice undefined.

**Figure 2:**
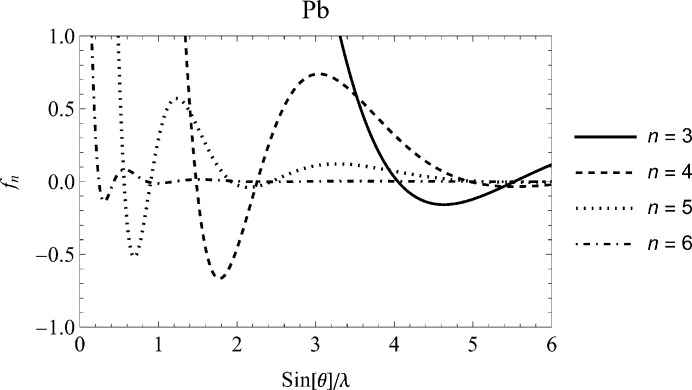
In the RTAB database (Kissel, 2000 ▸) the buildup of the atomic form factor is based on summing the contributions from atomic shells defined by the principal quantum number *n*. The figure displays the contributions from 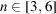 in the case of lead (Pb, *Z* = 82). An ordinate window of 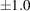 is chosen to emphasize the oscillating behaviour.

Altogether, it seems worthwhile to examine modelling of X-ray atomic form factor data once more. The key to the present approach is found in Appendix C, formula (C16), in the textbook by Kirkland (2010 ▸), in the use of the inverse Mott–Bethe formula (Mott & Bragg, 1930 ▸; Bethe, 1930 ▸; Bethe & Jackiw, 1986 ▸) as analytical model. It is revealed that this construction, with the electron scattering factor expressed by a sum of Gaussians, may partly deal with the inherent oscillating behaviour of 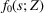 (see Fig. 2 ▸). By examining a series of tabulations of X-ray atomic form factors, it became evident that this approach works satisfactorily for most cases from the Hartree–Fock atomic form factors by Cromer & Mann (1968*a* ▸) to the recent Dirac–Hartree–Fock calculations by Olukayode *et al.* (2023 ▸).

## 2. Formulas

In this short survey the subscript X indicates X-rays while e indicates electrons (otherwise the subscript 0, selected to indicate zeroth order in the scattering factor, is used throughout for X-rays). We quote the following formula for X-ray elastic scattering, 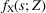 , in the form factor approximation (Kissel & Pratt, 1985 ▸), 

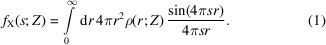

Here 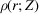 is the electron number density for element *Z* (assumed to be spherically symmetric).

The inverse Mott–Bethe equation, which is outlined within the framework of non-relativistic quantum mechanics (Bethe & Jackiw, 1986 ▸), gives a link between the X-ray and the electron form factors, 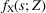 and 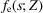 , respectively, 

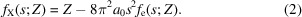

Here 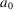 is the Bohr radius.

Analytical models of impact for this work are as follows:

(i) The sum of *n* Gaussians normally incorporating a constant term, here denoted as 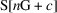 , 

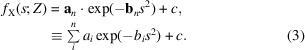

The formulation of equation (3) with an *n*-dimensional coefficient vector, 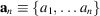 , and a corresponding vector of Gaussian basis functions, 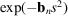 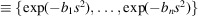 , is especially efficient for numerical calculations. Generally 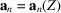 , 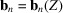 , but the *Z* dependence is normally not explicitly denoted.

(ii) The exponential or logarithmic polynomial model (in nonlinear least-squares calculations the two formulations may lead to slightly different results), denoted 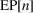 or 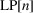 : 

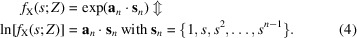

The model 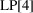 was used by Fox *et al.* (1989 ▸) to analyse data in the *s* range 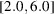 Å^−1^, *cf*. Maslen *et al.* (1992 ▸).

(iii) The inverse Mott–Bethe equation with the electron form factor expressed by summing *n* Gaussian terms (the number *n* may depend upon *Z*). A constant, α, is included as well. The model is denoted by 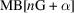 . It is emphasized that it is the analytical property of the generic term, 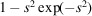 , which is important, as it gives rise to a curvature that locally may model part of an oscillation. Equation (5) works to model X-ray atomic form factor data defined for a finite range in *s* ( 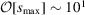 Å^−1^):

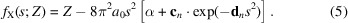

This formula is also applicable in an analysis of X-ray form factors of ions in which case *Z* is interpreted as the number of electrons, *cf*. Section 5. In fact, equation (5) is a limit of another model: 

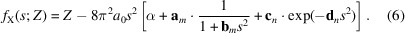

This model, incorporating *m* Lorentzian and *n* Gaussian basis functions, is symbolized by 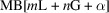 . The model 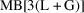 has been examined by Kirkland (2010 ▸). This class of models have been tested, but not found appropriate for the data material examined, *cf*. Section 5. One should also mention an expression built by a sum of Lorentzians and their squares: 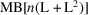 . An asymptotic version (having 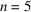 ), designed to cover the complete range 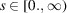 Å^−1^, is analysed in the work by Lobato & Van Dyck (2014 ▸). Since here we deal with exclusively truncated *s* ranges, this case is not explored further.

A single MB model of type (iii) equation (5) is recommended as an analytical representation of the X-ray atomic form factor for a given element *Z* whenever data are given in a finite range of 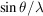 .

## 3. Method

The calculations were performed using the *Mathematica* function NonlinearModelFit (Wolfram Research, 2022 ▸). It returns a symbolic FittedModel object representing the nonlinear model that has been constructed. All observations are associated with unit weights. We may categorize the complete procedure in the following steps:

*Search*. A built-in random-number generator is applied to obtain initial values in the refinement process for the *d* parameters. RandomReal[{*x*_min_, *x*_max_}] chooses reals with a uniform probability distribution in the range 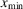 to 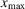 . This is an approach also applied in other works (*cf*. Peng *et al.*, 1996 ▸). The first stage normally involves six Gaussians, *i.e.* 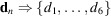 with (all *d* parameters are expressed in the unit Å^2^):

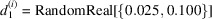




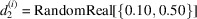




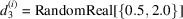




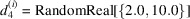




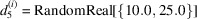




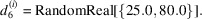




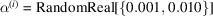
 , while for the *c* parameters the default value, 1, is used for startup. A nonlinear model is constructed without any *a priori* parameter constraints. A search typically consists of 100 repetitions of the refinement process, each starting with a different set of random parameters. For a model to be accepted after refinement, the following conditions are imposed on its parameters: 

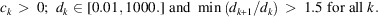

They effectively prevent results that cannot be further processed and have emerged from a growing experience.

*Repair*. In the case of a missing outcome for element 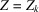 in the search process, one may use the full parameter set obtained for another element, 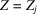 , as initial values in a single refinement: 

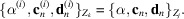

Normally 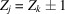 .

*Expand*. The complete search process spans six to nine Gaussians in the model 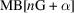 . To further expand the model, 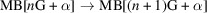 , the parameters 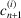 and 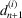 are arbitrarily set to 1.0 Å and 200. Å^2^, irrespective of the value of *Z*, and then added to the vectors 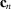 and 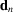 , 

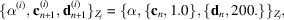

after which a single refinement is carried out. This approach has been very efficient and a dynamical change in the distribution of *d* values going from *n* to 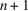 Gaussians is observed. *Expand* is repeated, sometimes after an intermediate stage where *Repair* is applied, until there is no further improvement, usually measured by the change in the value of the mean absolute error 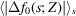 . This implies that the number of Gaussians in the model function may vary throughout the Periodic Table. Typically, the least number of Gaussians needed to obtain a value of the mean absolute error close to what may be expected from the precision of the published form factor data occurs for the noble gases and their preceding elements. With a growing number of parameters in the fitting process, the uncertainties in the refined parameters increase. Thus one has to individually assess as to when *Expand* should be interrupted. Furthermore, sudden striking changes in the value of the constant α may indicate that the model is pushed too far.

*Test*. A series of refinements are performed with small random changes in the *d* parameters [*e.g.* within ±(5–20)%]: 

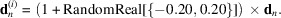

Usually 25–100 repetitions are carried out for each element. The level of acceptance is subject to the same general conditions as before and in addition the improvement of the mean absolute error should be significant, *e.g.* 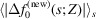 < 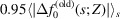 . This point is not especially crucial for models involving Gaussians only, but becomes essential in the search for a best fit when Lorentzians and Gaussians are combined in the model function [*cf*. equation (6)].

*Verify*. The least-squares process is always repeated once with the final parameters from the *Search*-and-*Expand* procedure as initial parameters, to ensure that a stable minimum in the refinements has been reached for all elements.

*Explore*. Plots of parameters versus atomic numbers are established to reveal any anomalies. Calculation of parameter uncertainties with separate assessments of the cases where the relative errors are larger than one is carried out. The behaviour of 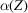 is specifically examined. Refinements resulting in 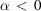 are normally not accepted {with the exception of Ir (*Z* = 77) and Pt (*Z* = 78) in data set ITC (see below for definition), both ascribed to the final model 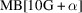 }. In most cases, unexpected deviations occur when too many parameters are incorporated into the model, and consequently the final parameter set may be reduced: 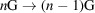 .

## 4. Analyses

The X-ray form factor data sets covered in this work are denoted as follows (entries marked with * have associated model functions as given in Table 1 ▸): CM (Cromer & Mann, 1968*a* ▸,*b* ▸)*; ITiv (Cromer & Waber, 1974 ▸)*; ITC (Maslen *et al.*, 1992 ▸; Waasmaier & Kirfel, 1995 ▸)*; WSSS (Wang *et al.*, 1993 ▸); SC (Su & Coppens, 1997 ▸)* (*cf*. http://harker.chem.buffalo.edu/group/ptable.html); Krf (Kissel, 2000 ▸); OFFV1 (Olukayode *et al.*, 2023 ▸)*; OFFV2 (Volkov, 2023 ▸).

For specific details of the quantum mechanical calculations leading to the electron number density, 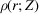 , and then to the X-ray form factor by applying equation (1), the original publications and the references therein should be consulted.


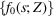
 are calculated on specific *s* grids for various sets of elements 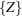 of the Periodic Table. The form factor data are published over a period of more than half a century and it is rather remarkable that a common construction of analytical representations works so well for all cases.

**Figure 3:**
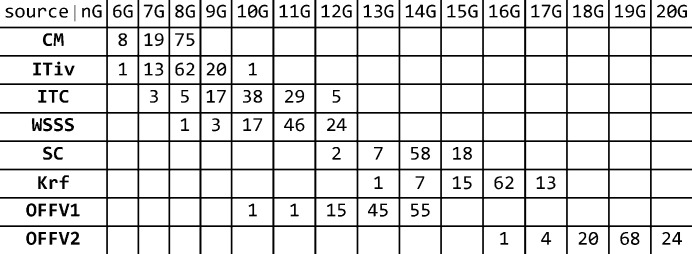
Number of elements with a parameter set involving 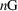 Gaussians.

The final analytical setup for each data set is comprised of model functions 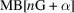 of equation (5). The number of basis functions involved is listed in Fig. 3 ▸. *n* spans the interval 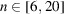 . Factors of importance for the least-squares fits are the number of data points, their precision and the sampling grid. These key figures are summarized below:

CM: the original data compilation is characterized by 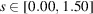 Å^−1^. 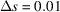 Å^−1^, in a total of 151 entries. 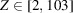 . Form factors are presented with a precision of 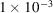 .

ITiv: in this compilation 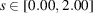 Å^−1^, in a grid 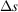 : 0.00 (0.01) 0.20; 0.20 (0.02) 0.50; 0.50 (0.05) 0.70; and 0.7 (0.1) 2.0 Å^−1^ + {0.25, 0.35, 0.45} Å^−1^. Thus there are 56 data entries for each element *Z*, 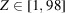 . The data precision is 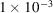 .

ITC: here 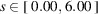 Å^−1^. The data in ITiv have here been extended by the entries at 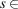 {2.50, 3.00, 3.50, 4.00, 5.00, 6.00} Å^−1^. This extension was partly conducted by Doyle & Turner (1968 ▸) in a genuine quantum mechanical calculation and partly by Fox *et al.* (1989 ▸) applying polynomial curve fitting and extrapolation to fill the gaps left by Doyle & Turner. In total there are 62 entries here denoted as the IUCr grid. ITC also presents X-ray form factors for the elements 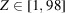 with a precision 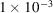 .

WSSS: 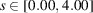 Å^−1^, in a grid 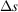 : 0.000 (0.025) 0.500; 0.500 (0.050) 1.000; 1.000 (0.100) 3.000; and 3.000 (0.200) 4.000 Å^−1^. Thus there are 56 data entries for each element *Z*, 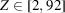 . The precision is a variable as 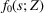 is given with five significant digits in a decimal form.

SC: here 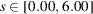 Å^−1^, 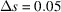 Å^−1^, in a total of 121 entries. 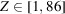 . Precision is set to 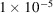 when 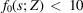 and 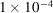 when 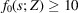 . Notice that for Si (14), P (15) and S (16) 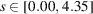 Å^−1^, while for La (57) 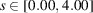 Å^−1^.

Krf: form factors are extracted from the RTAB database (*cf*. https://starship.org/RTAB/RTAB.php) entry data_RF. They are truncated to the range 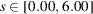 Å^−1^. Here 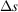 varies among the elements and the number of entries amounts to 143–507. *Z* spans the interval 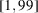 . The precision is also a variable as 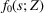 , of order 10^−6^–10^1^, are stored with eight significant digits in scientific format.

OFFV1(2): the most recently published data. In fact there are two versions: OFFV1 given in the supporting information file ae5122sup4.txt of Olukayode *et al.* (2023 ▸). Here 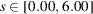 Å^−1^ with 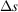 given by the IUCr grid. 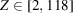 and the precision is 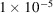 . A more complete set generated by the same authors, OFFV2, has been provided by Volkov (2023 ▸). Specifications: 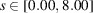 Å^−1^, 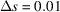 Å^−1^, in a total of 801 entries for each element. All form factors are presented with ten digits after the decimal point.

**Figure 4:**
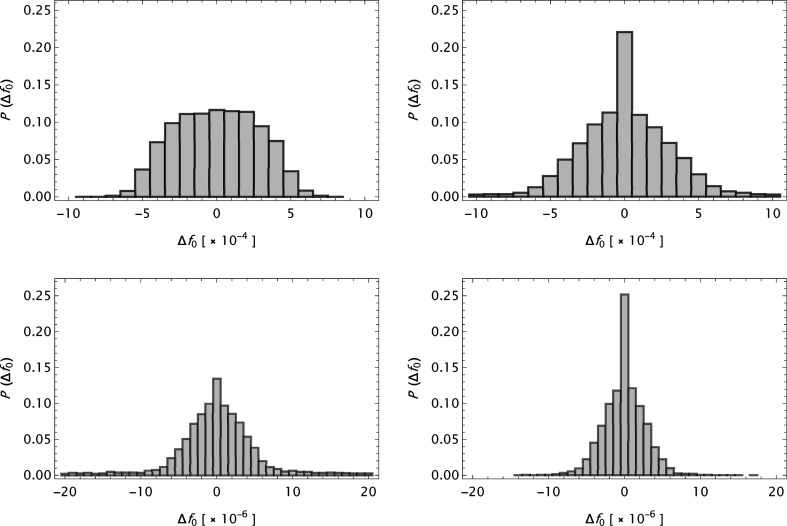
Histograms of 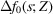 . The bin heights are given by relative numbers. Upper left: CM. Upper right: ITC. Lower left: SC. Lower right: OFFV1.

**Figure 5:**
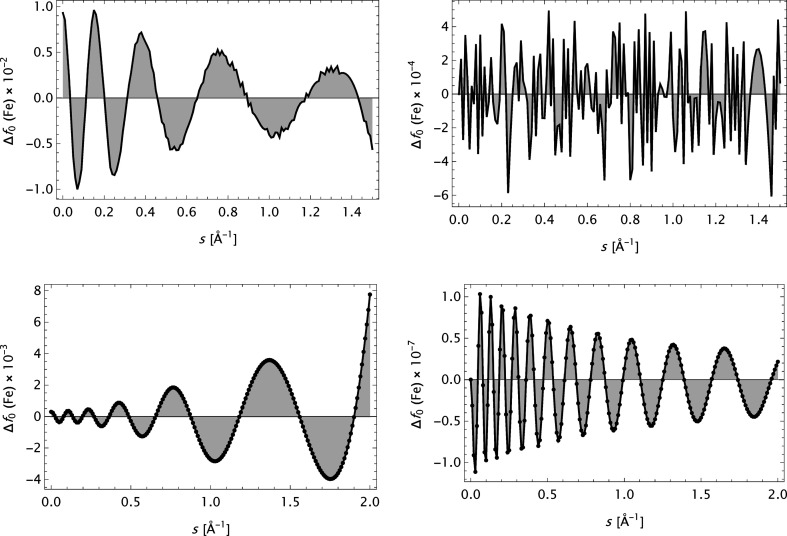
The error 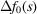 for iron (Fe). Upper row: CM data. Left: model 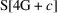 . Right: model 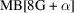 . Lower row: OFFV2 data. Left: model 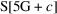 . Right: model 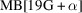 .

**Figure 6:**
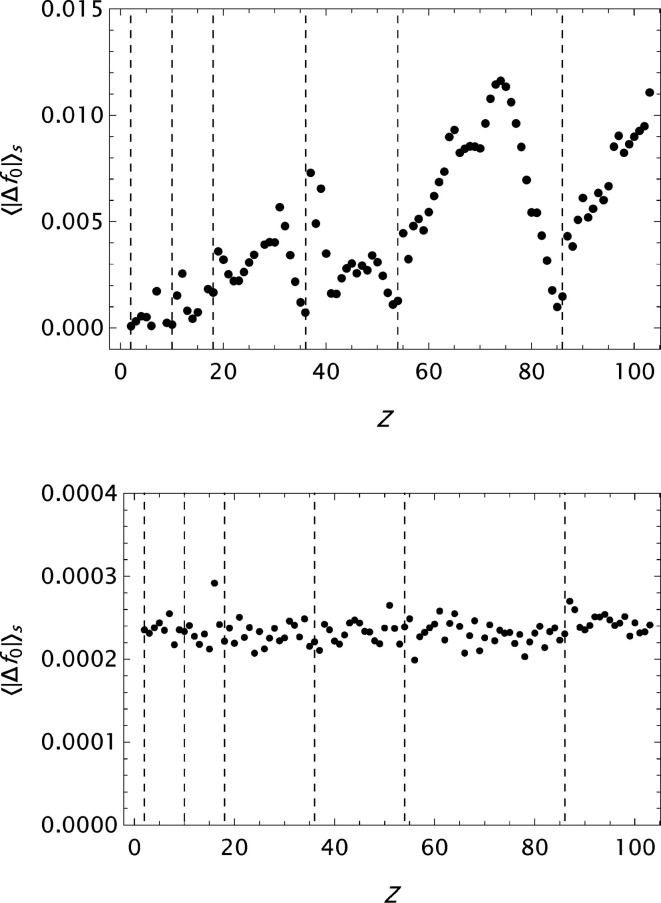
CM: the mean absolute error 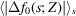 as a function of *Z*. Top: original result by Cromer & Mann (1968*a* ▸). Bottom: result obtained by the present approach.

**Figure 7:**
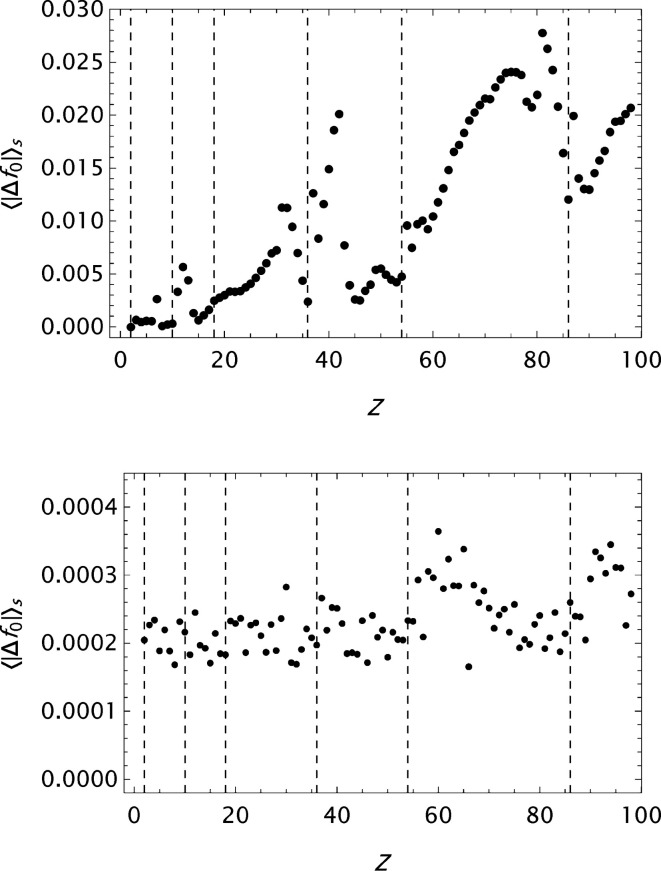
ITiv: the mean absolute error 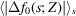 as a function of *Z*. Top: original result by Cromer & Waber (1974 ▸). Bottom: result obtained by the present approach.

**Figure 8:**
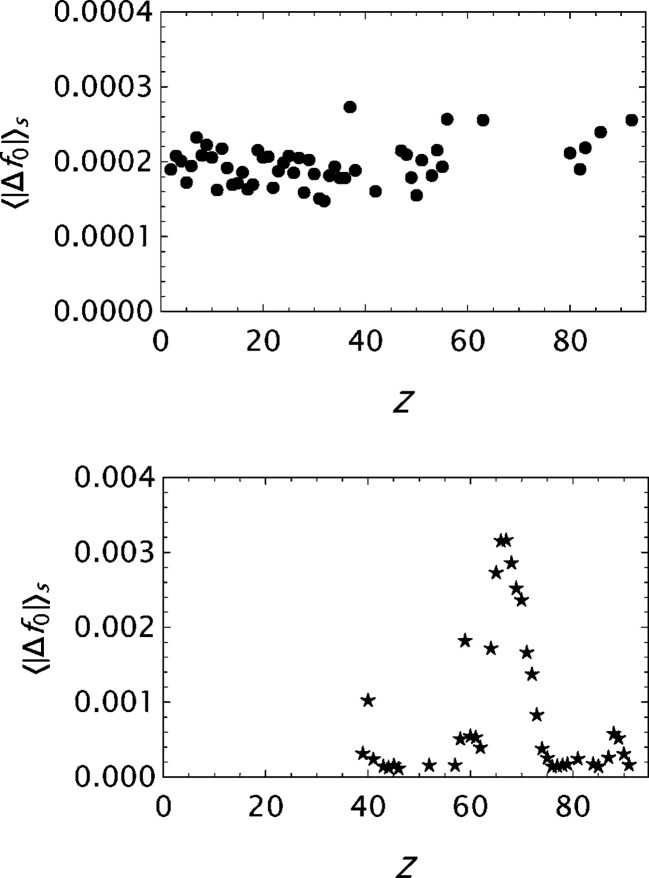
ITC: the mean absolute error 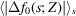 as a function of *Z* for the atomic form factor data compiled by Maslen *et al.* (1992 ▸) obtained by applying the new modelling function. Top: for elements where 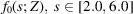 Å is given by Doyle & Turner (1968 ▸). Bottom: for elements where 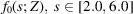 Å is given by Fox *et al.* (1989 ▸).

Table 3 ▸ summarizes the statistical measures 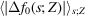 and 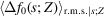 (where r.m.s. is root-mean-square) for the complete data sets. ( 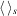 indicates an average over all *s* with fixed *Z*, while 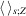 indicates an average over all *s* and *Z* values. Elements *Z* = 14–16, 57 in SC, published with an *s* range different from the others, were discarded in this calculation. For ITC a special selection was made, see the main text.) For CM, ITiv and ITC the corresponding measures (original versus new) obtained by the 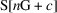 model using parameters from Table 1 by Cromer & Mann (1968*a* ▸), Table 2.2B by Cromer & Waber (1974 ▸) and Table 1 by Waasmaier & Kirfel (1995 ▸) have been included. Furthermore 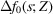 values are presented in histograms for four data sets in Fig. 4 ▸. The new analytical model results in a substantial improvement in the fits to the tabulated form factors. One is more or less approaching the limits set by the precision in the original data compilations. However, while the analysis of 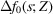 for ITiv data seems to have reached a random state, the OFFV2 data still exhibit an oscillatory behaviour (see Fig. 5 ▸). Figs. 6 ▸ ▸ ▸ ▸ ▸ ▸ ▸–13 ▸ summarize 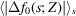 as a function of *Z* for all cases studied. For WSSS to OFFV2 plots of 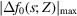 are included. Special attention should be paid to the ITC analysis presented in Fig. 8 ▸. The data in Table 6.1.1.1 (Maslen *et al.*, 1992 ▸) are compiled from various sources. The main part 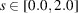 Å^−1^ is identical to Table 2.2.A (Cromer & Waber, 1974 ▸), while the extensions to include 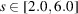 Å^−1^, as mentioned above, are built based on two very different approaches. This is reflected in the refinements as the elements having an *s* extension by Fox *et al.* (1989 ▸) have a different signature from the data with extensions supplied by Doyle & Turner (1968 ▸). Fig. 2 in the paper by Fox *et al.* (1989 ▸) reveals that a polynomial fitting to 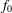 (3.0 Å^−1^; *Z*), having relatively large gaps in *Z*, may lead to less accurate values than expected from the precision in their presentation. To emphasize this point 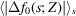 as a function of *Z* has been presented in two separate parts in Fig. 8 ▸. The statistical properties for ITC, given in Table 3 ▸, are calculated for 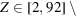 {40, 59, 64–73}. One should also mention that the values for the mean absolute error, 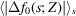 , as presented in Fig. 7 ▸ using the original 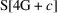 model, differ from what is found in Table 2.2.B by Cromer & Waber (1974 ▸) (maximum absolute errors are however reproduced). It may be that the values presented by Cromer & Waber (1974 ▸) are calculated based on an *s* grid different from that reported (*cf*. Cromer & Waber, 1964 ▸). Figs. 14 ▸ and 15 ▸ depict the *Z* dependence for α and *d*_1_–*d*_4_ for some selected stages in the analysis. Clearly, in these cases, α is a well behaved parameter, its value depends upon the actual *s* span and it is typically highly correlated to 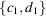 . We also observe that the lowest *d* values are nearly insensitive to the *Z* values, but depend on the *s* grid and the precision of the raw form factor data.

Notice that, in most of the figures having *Z* as independent variable, the positions of filled shells associated with the principal quantum numbers are indicated with dashed vertical lines. Particularly in the initial parts of the *Search*-and-*Expand* procedure explicit parameter and error variations within a shell (as functions of *Z*) are observed.

The form factor compilation OFFV2 is in many respects the most complete. It has a large span, very fine grid and high precision. Some aspects regarding the final set of parameters in the analytical models for these data are graphically presented in Figs. 16 ▸ ▸–18 ▸. In the expansion of the model it is observed that 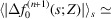 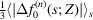 . Here superscript 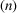 represents the number of Gaussians in the model. Thus expanding the model eight times after *Search* leads to a reduction of the mean absolute error by a factor 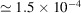 .

## 5. Discussion

The first step in this study was to analyse the atom form factor data by Kirkland, trying to expand his analytical model 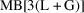 into 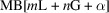 . This did not progress as smoothly as expected. The best fits were finally achieved for models 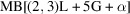 with two Lorentzians for 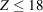 . However, the improvements of 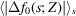 were not substantial. Fig. 19 ▸ depicts the *Z* dependence of the mean absolute error both for the analytical model developed by Kirkland and the present approach. A detailed study, here exemplified by 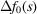 evaluated for iron (*cf*. Fig. 20 ▸), may explain the reason for the behaviour. The fine ripples, superimposed upon the type of oscillating background normally encountered, which is observed in the difference plots, are assumed to prevent a normal development of the refinements by *Expand*.

The model 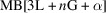 has been examined in connection with most of the form factor data sets. It behaves differently compared with 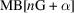 . Including Lorentzian functions seems to give rise to a more complex parameter space where many different parameter combinations lead to almost identical values for 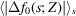 . Thus it becomes difficult to verify whether a global minimum is really reached. Repeated cycles of *Tests* must then be carried out until no better fits are deduced. The *Expand* procedure does neither function as efficiently as in the pure Gaussian case as the subsequent refinements may follow a path between local minima and miss the global one. Restrictions on the sign of the coefficients of either the Lorentzian or the Gaussian basis functions must be abandoned and the close-packed local minima often involve different sign combinations of the coefficients. Altogether, using model 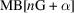 in the refinements leads smoothly to reproducible results and is the preferred choice.

In the RTAB database the Krf data span the range 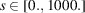 Å^−1^ which is truncated to 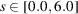 Å^−1^ to be comparable with the range found in most form factor publications. The parameters associated with the analytical model refined for this range may be used as initial parameters for a data set increased to incorporate *s* values up to and including 7.0 Å^−1^. This procedure is then continued in steps of 1.0 Å^−1^ until a span 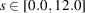 Å^−1^ is reached, which in many respects represents an upper limit in range. In this process 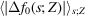 increases in each step in total by a factor of ∼10. To regain approximately the value found for the original range, the model must be expanded. 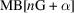 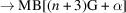 is sufficient. To model atomic form factor data determined for an infinite range, one must search for other analytical models than the present one.

**Figure 13:**
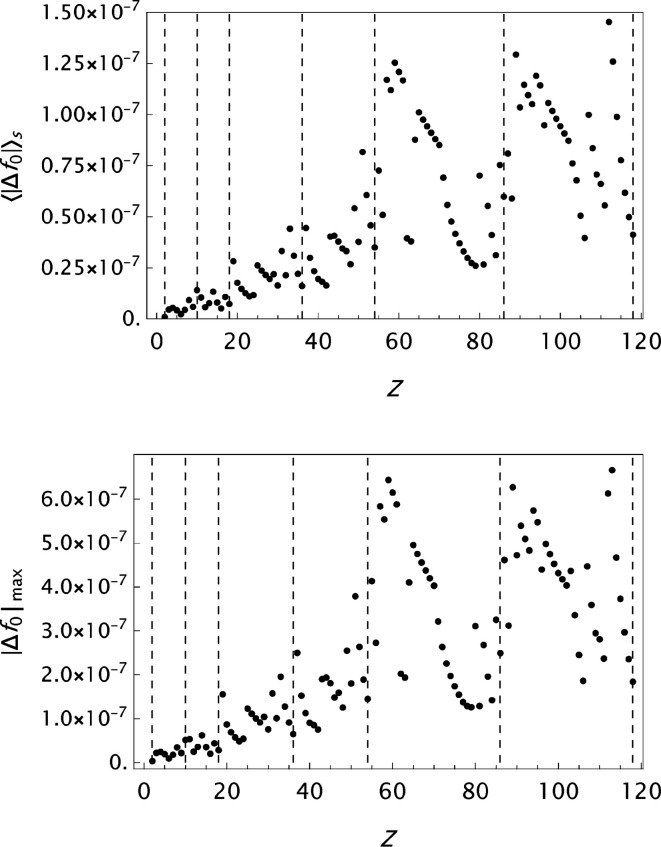
Analysis of the OFFV2 data generated by Olukayode *et al.* (2023 ▸) and made available by Volkov (2023 ▸). Results for the final 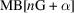 parametrizations. Top: the mean absolute error 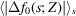 as a function of *Z*. Bottom: the maximum absolute error 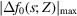 as a function of *Z*. The *Z* variation indicates that the statistical limit set by the data precision is not yet reached.

**Figure 14:**
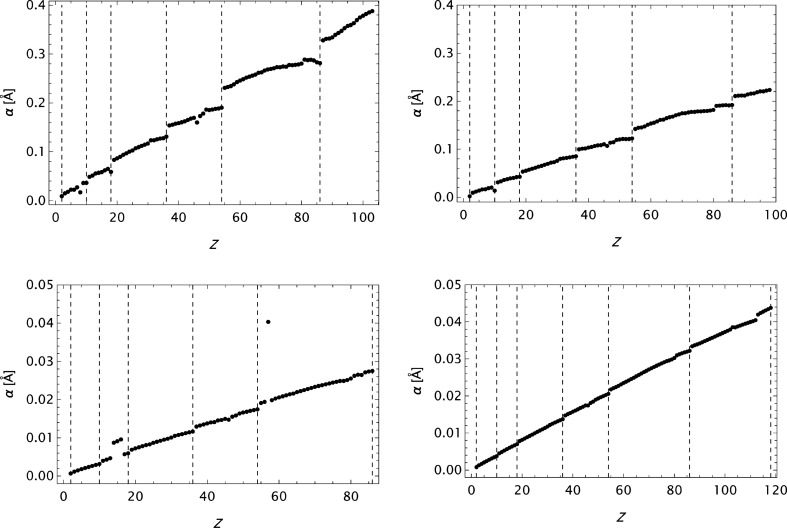
Plots of 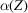 . Upper left: CM data; model 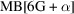 . Upper right: ITiv data; model 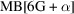 . Lower left: SC data; model 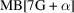 . Lower right: OFFV1 data; model 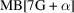 .

**Figure 16:**
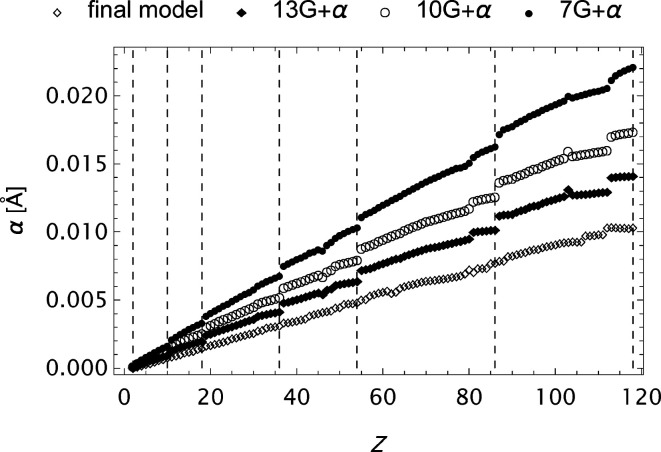
The OFFV2 data; α as a function of *Z* at various stages of the refinement process.

**Figure 19:**
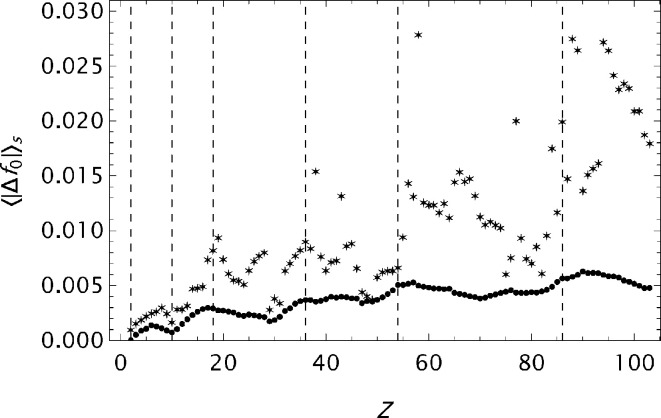
The mean absolute error 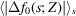 as a function of *Z*. Atomic form factors calculated by Kirkland. The * symbols are associated with model 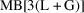 with parameters given by Kirkland. The other symbols are associated with model(s) 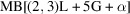 .

**Figure 20:**
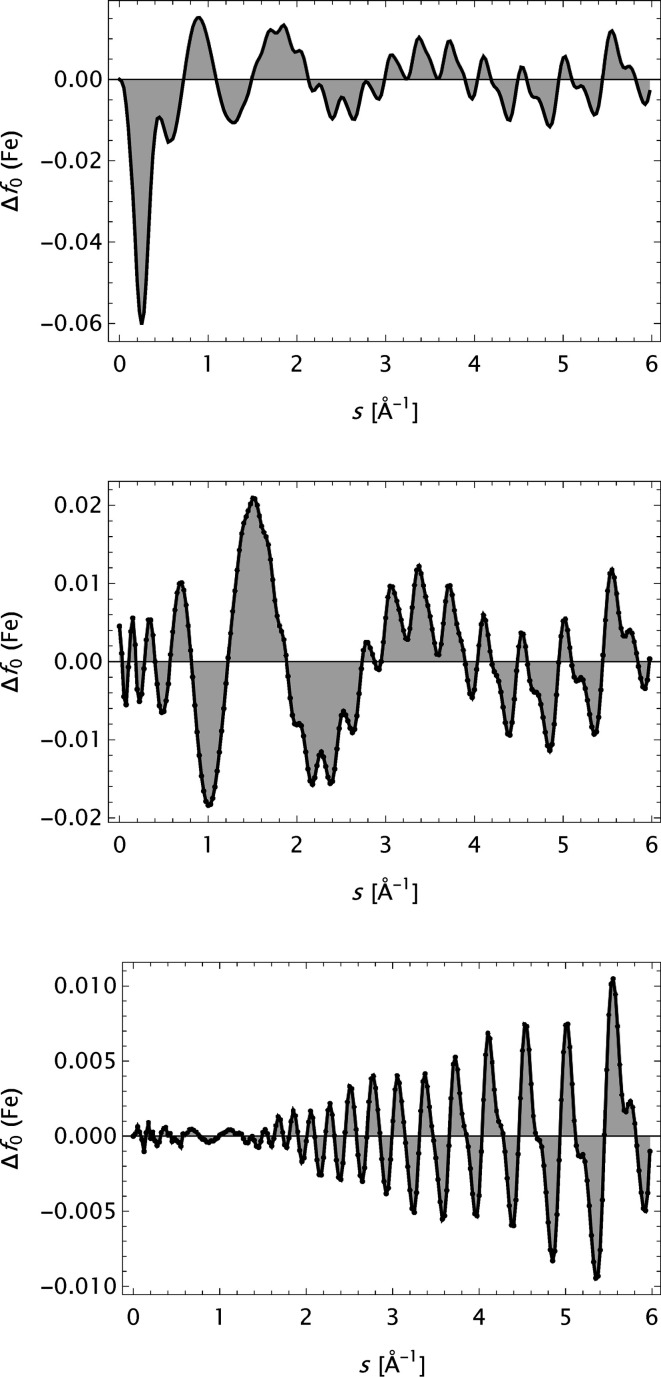
The deviation 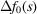 for Fe calculated based on form factor data by Kirkland. Top: original 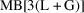 model function. Middle: 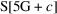 model function. Bottom: 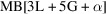 model function.

**Figure 21:**
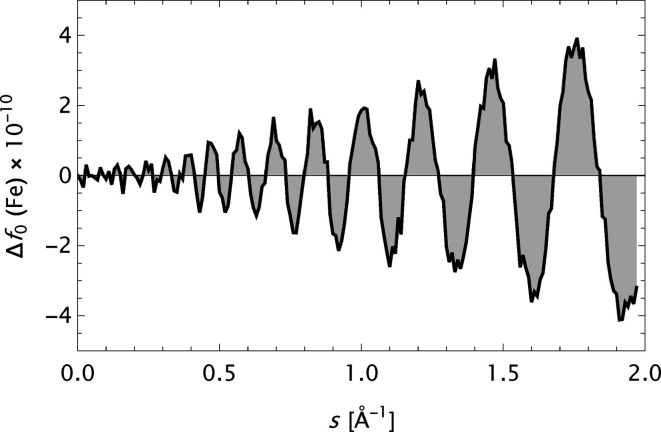
The error 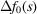 for Fe for the ultimate final model 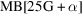 for OFFV2 data. Here shown for the range 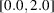 Å^−1^ for easy comparison with Fig. 5 ▸ (lower row – right).

Fig. 13 ▸ indicates that it should be possible to push the model even further for the high-quality OFFV2 data. When 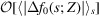 is approaching 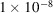 downwards, one has to increase the values of the internal parameters MaxIterations, PrecisionGoal and AccuracyGoal in the *Mathematica* function NonlinearModelFit to obtain a reliable fit. In addition, when more Gaussians are incorporated in the model, the *d* values tend to pack more closely and the condition of a minimum ratio for neighbouring values of 1.5 must be relaxed. Altogether these adjustments cause the computing time of a refinement to increase considerably. Here form factor data for Fe have been examined and it has been possible to increase in steps the number of Gaussians from 19 to 25 (*cf*. Fig. 21 ▸), and thereby reduce the mean absolute error from 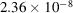 to 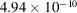 , still an order of magnitude larger than the actual statistical limit for data with ten digits’ precision. It may be appropriate to discuss whether such a level of accuracy in the original data and in the modelling is ever needed. In X-ray diffraction studies one has to take into account effects due to non-spherical parts of the electron-density distribution and dispersive parts of the scattering process. This will affect what should be regarded as the relevant significant digits of X-ray atomic form factor data.

**Figure 22:**
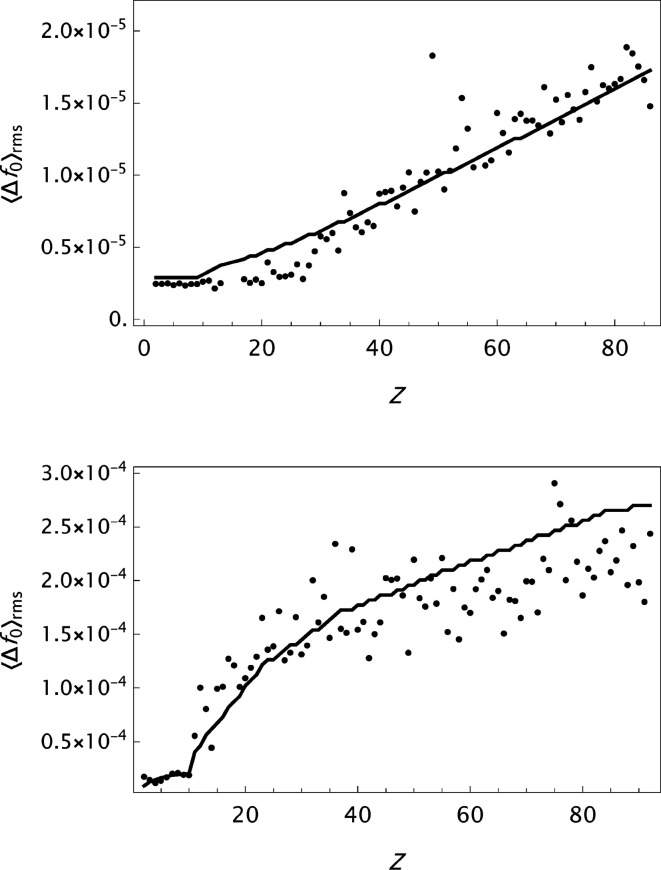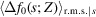 for WSSS data (top) and SC data (bottom). The guiding lines are calculated from the simple model of equation (7) with 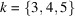 for WSSS and 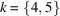 for SC.

Assuming that the deviations, 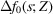 , have a uniform distribution [the standard deviation for a uniform distribution of width 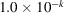 is 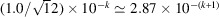 ] determined by the precision of the observations, the following formula estimates the r.m.s. value 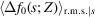 (evaluated on the *s* grid): 

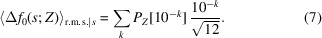




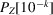
 is the relative number of the form factors for element *Z* with precision 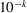 . Equation (7) is applied in connection with WSSS and SC data and the outcomes are depicted in Fig. 22 ▸. Apparently, one is close to the statistical prediction, which confirms that high-quality fits to the observations have been obtained.

A preliminary analysis of form factors for the ions F^−^, Na^+^, Mg^2+^, using the 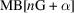 model, was undertaken based on data in Table 4 by Wang *et al.* (1996 ▸). The precision of these data is 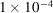 . The results for the mean and maximum absolute errors are reported in Table 4 ▸. Also, for these cases the final analytical models reproduce the data very well.

## 6. Concluding remarks

An analytical model based on the inverse Mott–Bethe relationship, parametrized as a sum of Gaussians, and denoted as 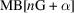 , has proved to be a straightforward, refinable and well behaving function to represent X-ray atomic form factor data. From the outset, one should allow a variable number of Gaussians in the model linked to the position of the elements in the Periodic Table. Form factor data calculated on a fine uniform grid and to a high precision lead through the refinement of the model parameters to a set of functions that reproduces the input data to an unprecedented high accuracy. This, together with its straightforward implementation, make models of type 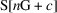 and 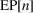 obsolete. Ordering of the parameters by increasing exponents throughout the analysis has been of immediate importance in building the final models.

The challenges encountered working with the ITC form factor tables suggest that in forthcoming publications of the *International Tables for Crystallography*, these tables should be revised and brought to a self-consistent level. The data by Olukayode *et al.* (2023 ▸) seem to be a strong candidate. As a by-product, elastic atomic scattering factors of electrons may be directly deduced from this modelling of X-ray form factors.

All final 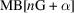 parameter sets obtained are available as supporting information.

## Supplementary Material

Parameters of the analytical model for CM data. DOI: 10.1107/S2053273323003996/ae5130sup1.txt

Parameters of the analytical model for ITiv data. DOI: 10.1107/S2053273323003996/ae5130sup2.txt

Parameters of the analytical model for ITC data. DOI: 10.1107/S2053273323003996/ae5130sup3.txt

Parameters of the analytical model for WSSS data. DOI: 10.1107/S2053273323003996/ae5130sup4.txt

Parameters of the analytical model for SC data. DOI: 10.1107/S2053273323003996/ae5130sup5.txt

Parameters of the analytical model for Krf data. DOI: 10.1107/S2053273323003996/ae5130sup6.txt

Parameters of the analytical model for OFFV1 data. DOI: 10.1107/S2053273323003996/ae5130sup7.txt

Parameters of the analytical model for OFFV2 data. DOI: 10.1107/S2053273323003996/ae5130sup8.txt

## Figures and Tables

**Figure 9:**
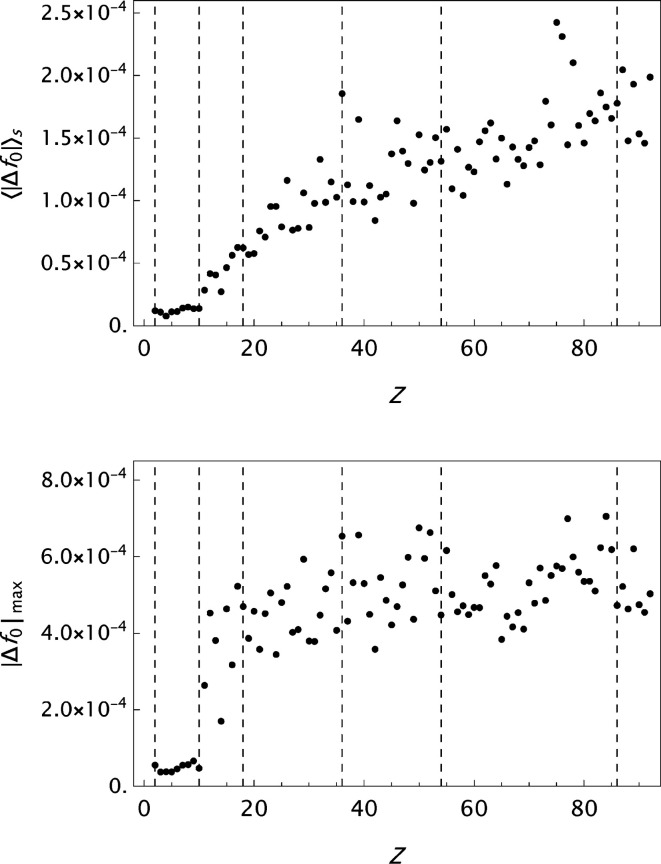
WSSS: analysis of data published by Wang *et al.* (1993 ▸). Results for the final 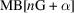 parametrizations. Top: the mean absolute error 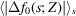 as a function of *Z*. Bottom: the maximum absolute error 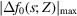 as a function of *Z*.

**Figure 10:**
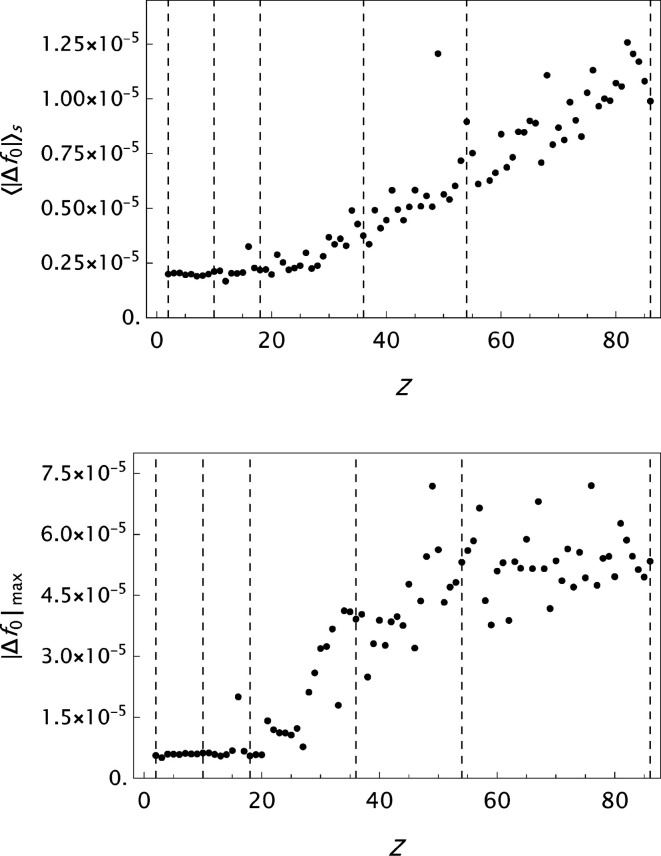
SC: analysis of data published by Su & Coppens (1997 ▸). Results for the final 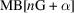 parametrizations. Top: the mean absolute error 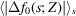 as a function of *Z*. Bottom: the maximum absolute error 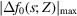 as a function of *Z*.

**Figure 11:**
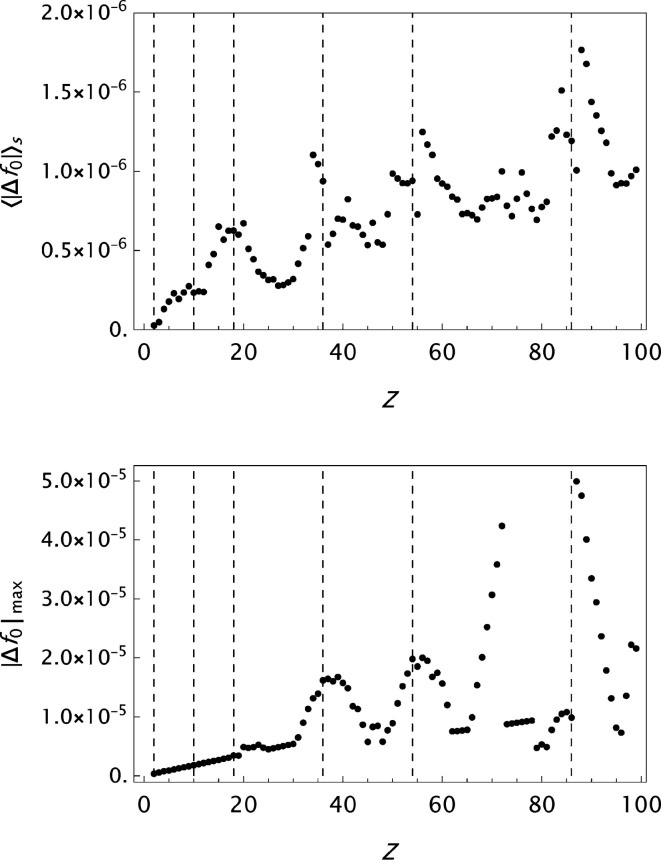
Krf: analysis of data published by Kissel (2000 ▸). Results for the final 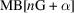 parametrizations. Top: the mean absolute error 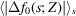 as a function of *Z*. Bottom: the maximum absolute error 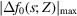 as a function of *Z*.

**Figure 12:**
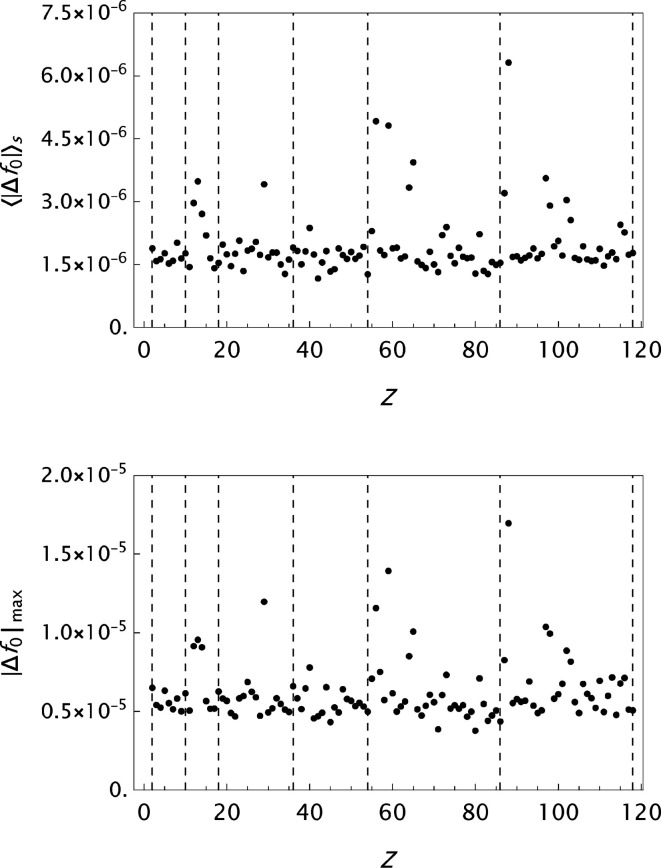
Analysis of the OFFV1 data published by Olukayode *et al.* (2023 ▸). Results for the final 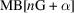 parametrizations. Top: the mean absolute error 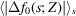 as a function of *Z*. Bottom: the maximum absolute error 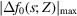 as a function of *Z*.

**Figure 15:**
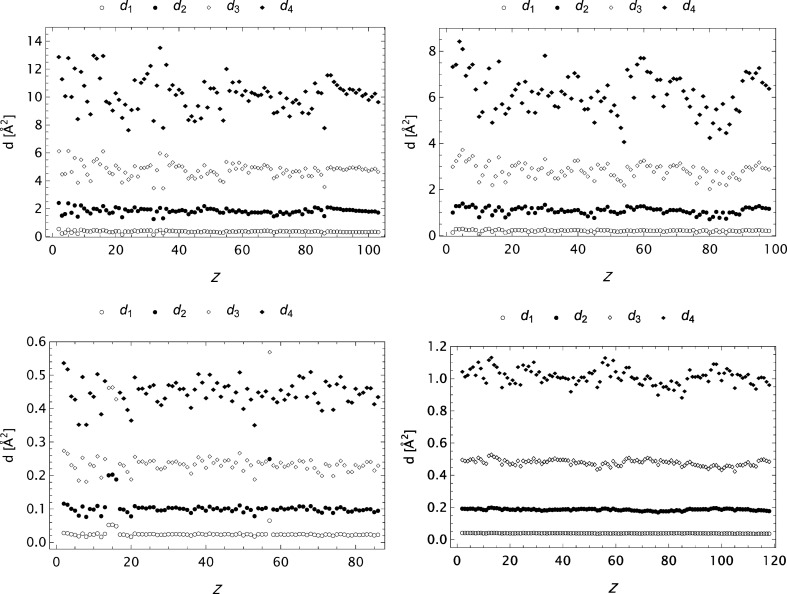
Plots of 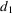 to 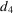 for some final models as functions of *Z*. Upper left: CM data. Upper right: ITiv data. Lower left: SC data. Lower right: OFFV1 data.

**Figure 17:**
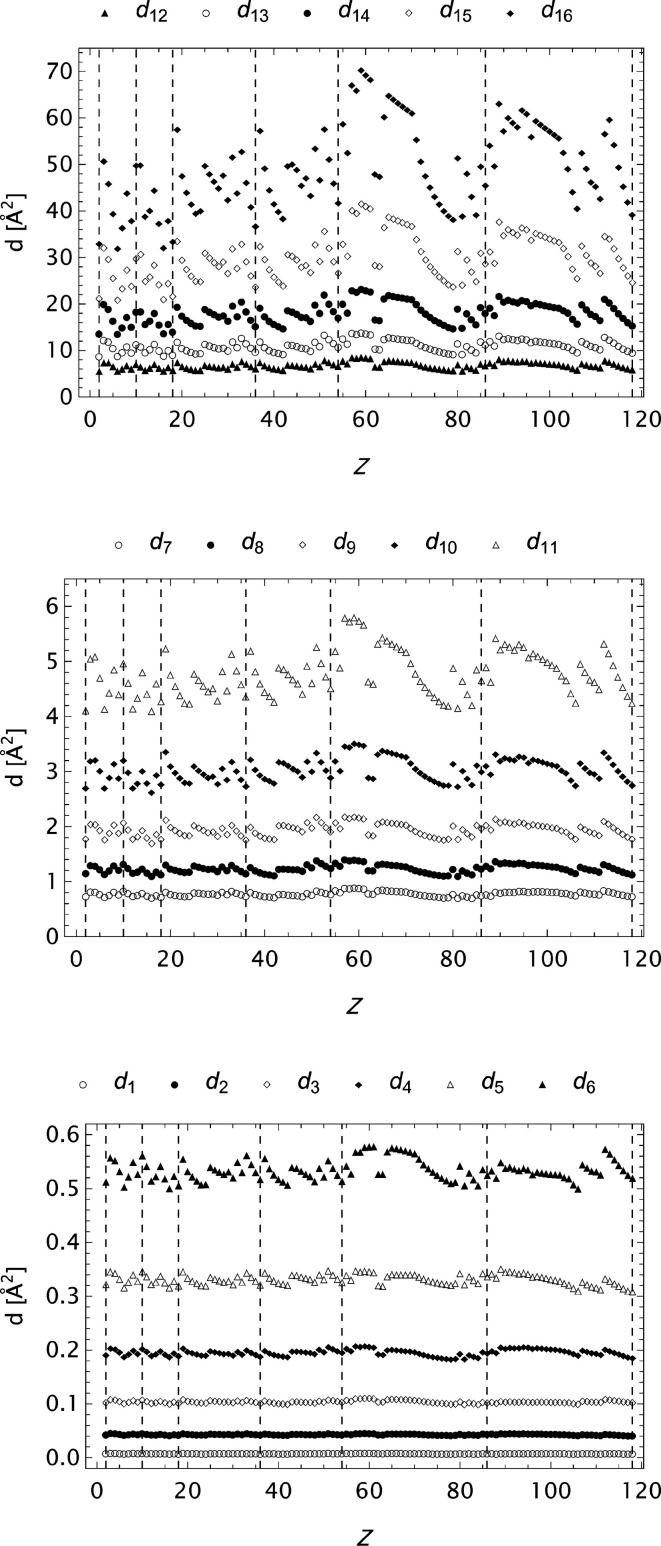
The extended OFFV2 data; 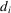 as a function of *Z*.

**Figure 18:**
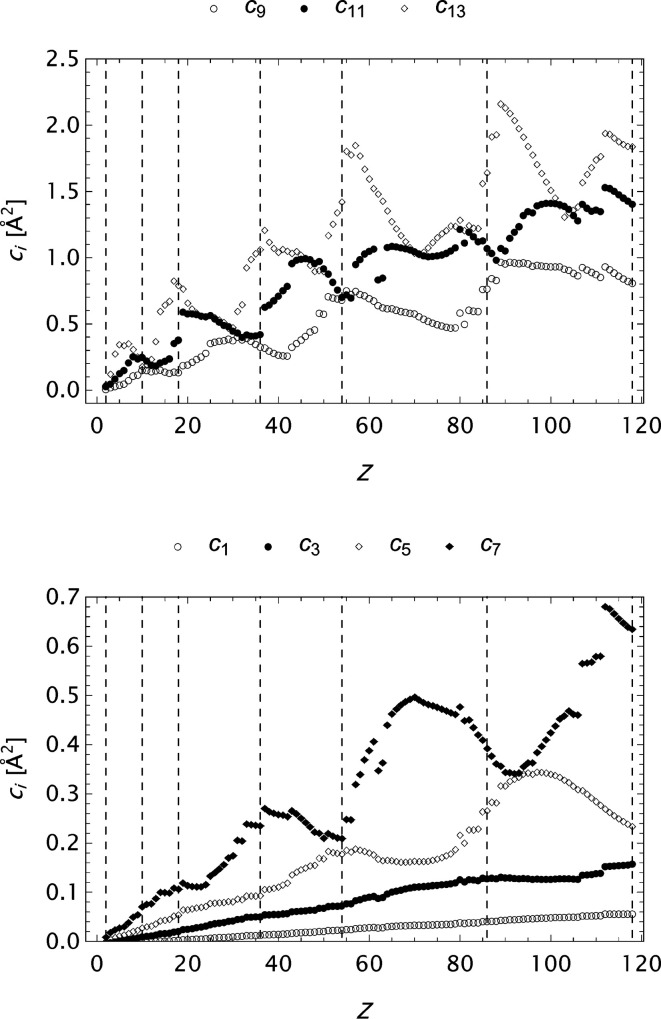
The extended OFFV2 data; 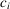 as a function of *Z*. Every other coefficient is included to obtain suitable resolution.

**Table 1:** Major compilations with associated *s* ranges and model functions (*cf*. Section 2 for nomenclature)

Authors (data)	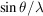 (Å^−1^)	Model
Cromer *et al.* (1964 ▸)	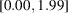	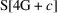
Cromer & Waber (1965 ▸)	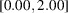	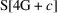
Cromer & Mann (1968*a* ▸)	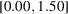	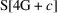
Doyle & Turner (1968 ▸)	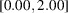	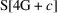
Lee & Pakes (1969 ▸) (Hanson *et al.*, 1964 ▸)	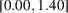 †	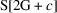
Hajdu (1972 ▸) (Tavard *et al.*, 1967 ▸)	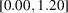	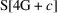
IT Vol. iv: Cromer & Waber (1974 ▸)	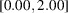	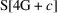
Fox *et al.* (1989 ▸) (Doyle & Turner, 1968 ▸)	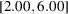	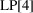
IT Vol. C: Maslen *et al.* (1992 ▸)	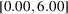 ‡	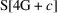 and 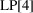
Rez *et al.* (1994 ▸)	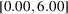 §	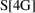
Waasmaier & Kirfel (1995 ▸) (Maslen *et al.*, 1992 ▸)	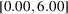	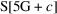
Su & Coppens (1997 ▸)	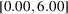 ¶	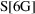
Kirkland (2010 ▸)	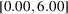	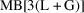
Lobato & Van Dyck (2014 ▸) (Kirkland, 2010 ▸)	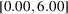	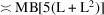
Olukayode *et al.* (2023 ▸)	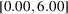 ‡	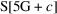 and 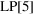 ††

† Mo *K*α radiation; 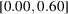 Å^−1^ for Cu *K*α.

‡ Split in two parts 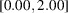 and 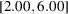 Å^−1^ with different model functions.

§ Two separate parameter sets, respectively, covering 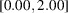 and 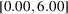 Å^−1^.

¶ Split in three equal parts with separate sets of parameters. Form factors for 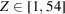 are analysed.

†† Parameters for 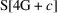 and 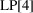 are also provided.

**Table 2:** Waasmaier & Kirfel parameters including uncertainties for *Z* = 66 (Dy) *a* and *c* are dimensionless quantities. 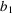 to 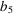 are sorted from top to bottom.

*a*	*b* (Å^2^)	*c*
88.69 (2833.73)	0.000665 (0.021645)	−83.28 (2834.18)
17.1 (0.4)	0.226 (0.007)	
26.67 (0.11)	2.28 (0.02)	
14.07 (0.12)	12.92 (0.16)	
2.77 (0.05)	122. (3.)	

**Table 3:** Statistical parameters

	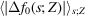	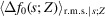
CM-original	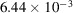	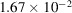
CM-new	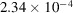	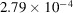
ITiv-original	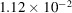	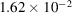
ITiv-new	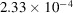	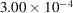
ITC-original	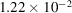	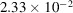
ITC-new	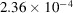	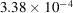
		
WSSS	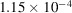	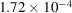
SC	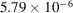	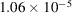
Krf	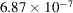	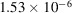
OFFV1	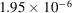	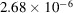
OFFV2	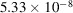	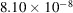

**Table 4:** A preliminary analysis of some ions Form factor data by Wang *et al.* (1996 ▸).

Ion	Model	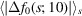	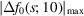
F^−^	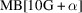	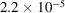	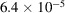
Na^+^	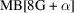	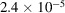	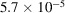
Mg^2+^	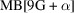	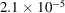	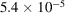
